# Low overlap between carbapenem resistant *Pseudomonas aeruginosa* genotypes isolated from hospitalized patients and wastewater treatment plants

**DOI:** 10.1371/journal.pone.0186736

**Published:** 2017-10-19

**Authors:** Andrej Golle, Sandra Janezic, Maja Rupnik

**Affiliations:** 1 National Laboratory for Health, Environment and Food, Maribor, Slovenia; 2 University of Maribor, Faculty of Medicine, Maribor, Slovenia; Purdue University, UNITED STATES

## Abstract

The variability of carbapenem-resistant *Pseudomonas aeruginosa* strains (CRPA) isolated from urine and respiratory samples in a large microbiological laboratory, serving several health care settings, and from effluents of two wastewater treatment plants (WWTP) from the same region was assessed by PFGE typing and by resistance to 10 antibiotics. During the 12-month period altogether 213 carbapenem-resistant *P*. *aeruginosa* isolates were cultured and distributed into 65 pulsotypes and ten resistance profiles. For representatives of all 65 pulsotypes 49 different MLSTs were determined. Variability of clinical and environmental strains was comparable, 130 carbapenem-resistant *P*. *aeruginosa* obtained from 109 patients were distributed into 38 pulsotypes, while 83 isolates from WWTPs were classified into 31 pulsotypes. Only 9 pulsotypes were shared between two or more settings (hospital or WWTP). Ten MLST were determined for those prevalent pulsotypes, two of them (ST111 and ST235) are among most successful CRPA types worldwide. Clinical and environmental carbapenem-resistant *P*. *aeruginosa* strains differed in antibiotic resistance. The highest proportion of clinical isolates was resistant to piperacillin/tazobactam (52.3%) and ceftazidime (42.3%). The highest proportion of environmental isolates was resistant to ceftazidime (37.1%) and ciprofloxacin (35.5%). The majority of isolates was resistant only to imipenem and/or meropenem. Strains with additional resistances were distributed into nine different patterns. All of them included clinically relevant strains, while environmental strains showed only four additional different patterns.

## Introduction

*Pseudomonas aeruginosa* is a typical opportunistic pathogen primarily causing nosocomial infections in immunocompromised patients, especially in intensive care units or in patients with other predisposing conditions [1,2]. As a nosocomial pathogen it is associated mainly with pneumonia, urinary tract infections and infections of skin and soft tissues. It is often isolated from respiratory tract of patients with chronic lung disease, including patients with cystic fibrosis [3–5]. *P*. *aeruginosa* genome codes for considerable number of enzymes and efflux pumps that contribute to intrinsic resistance towards different classes of antibiotics. Resistance is additionally acquired by mutation or horizontal gene transfers [2,6], thus *P*. *aeruginosa* has a high potential to develop multidrug resistance phenotype [4].

Because of different intrinsic or acquired mechanisms of resistance, *P*. *aeruginosa* infections are difficult to treat, and carbapenems are among the few available treatment options [7]. In recent years an increased prevalence of resistance to carbapenems among *P*. *aeruginosa* isolates started to emerge worldwide [8,9]. Carbapenem resistance in *P*. *aeruginosa* can be caused by loss of the porin OprD, overexpression of multidrug efflux pumps and bacterial production of carbapenemases [6,8]. Production of acquired carbapenemases is important mechanism of beta-lactam antibiotic resistance in *P*. *aeruginosa* in nosocomial as well environmental isolates and isolates with carbapenemases are usually resistant to all beta-lactams [10–12]. In Europe we encounter predominantly class B carbapenemases or metallo-beta-lactamases (VIM, IPM), while other types such as class A carbapenemases (KPC, GES) and class D carbapenemases (OXA) are rarely found.

In broader understanding of epidemiology of antibiotic resistant bacteria, their distribution and survival in other reservoirs, such as animals and environment, is also important. *P*. *aeruginosa* is ubiquitous and found in lakes, rivers, swimming pools, sewage, soil, animals, plants and plant detritus [2]. In the form of biofilm it can proliferate in municipal drinking water distribution systems [13]. Among different water environments, wastewater treatment plants (WWTPs) are considered an important hotspot for spreading of antibiotic resistances in different pathogens [14]. Resistant *P*. *aeruginosa* was described before in WWTP effluent and in the rivers downstream of WWTPs [15–17].

Carbapenem-resistant *P*. *aeruginosa* originating from patients and hospital environment could be potentially transferred with wastewater to WWTP. The aim of this study was to assess variability and antibiotic resistance profiles of carbapenem resistant *P*. *aeruginosa* strains isolated from hospitalized patients and WWTPs and to evaluate possible overlap between clinically relevant and environmental strains.

## Materials and methods

### Study settings

Clinical strains were selected from the carbapenem-resistant *P*. *aeruginosa* cultured from all respiratory and urine samples received by one of the laboratories within National laboratory for health, environment and food, NLZOH. This particular laboratory is providing diagnostic microbiological service for hospitals and other health care settings and general practitioners in the region serving population of 550.000.

Ethical approval for use of clinical samples was obtained by National Medical Ethic Committee (No. 92/03/14).

Two mechanical—biological wastewater treatment plants (WWTP) were selected for effluent sampling. Larger one, WWTP A, had in 2014 yearly inflow of approximately 11.000 x 10^6^ m^3^ of water and would collect community sewage water in region associated with hospital A. Smaller WWTP B located at a distance of 16 km from WWTP A had in 2014 yearly inflow of approximately 5.212 x 10^6^ m^3^ of water and would collect also community sewage water associated with hospital B.

Clinical and environmental samples were collected during the same period, from January to December 2014.

### Isolation, identification and antibiotic resistance testing of *P*. *aeruginosa* from clinical samples and selection of strains for the study

During the routine diagnostic procedures samples from respiratory tract were inoculated on blood agar (Biolife), chocolate agar (Oxoid) and ENDO agar (HImedia). Agar plates were incubated at 36°C for 24–48 hours. Urine culture was performed on cysteine electrolyte deficient agar (CLED), which was also incubated for 24–48 hours.

Identification of *P*.*aeruginosa* was performed by matrix-assisted laser desorption/ionization time-of-flight mass spectrometry (MALDI-TOF) (Microflex MALDITOF, Bruker, Daltonics) by standard protocol specified by manufacturer. Isolated *P*. *aeruginosa* strains were routinely submitted to antibiotic susceptibility testing by disc diffusion method according to EUCAST recommendation (EUCAST, 2014). Each isolate was tested against ceftazidime (CAZ, 30 μg), cefepime (FEP, 30 μg), piperacillin/tazobactam (TZP, 100/10 μg), imipenem (IPM, 10 μg), meropenem (MEM, 10 μg), ciprofloxacin (CIP, 5 μg), tobramycin (NN, 10 μg), gentamycin (GM, 10 μg), amikacin (AN, 10 μg) and netilmicin (NET, 10 μg). Quality control was carried out using *P*. *aeruginosa* (ATCC 27853) and *E*. *coli* (ATCC 25922).

*P*. *aeruginosa* cultured from respiratory tract samples or urine resistant to either imipenem or meropenem would be per laboratory protocol stored at -70°C. For this study the database of stored carbapenem resistant *P*. *aeruginosa* was screened and first isolate per patient was selected. Any subsequent isolate for a given patient was included only if the resistance profile differed from the first isolate from the same patient.

### Isolation and characterization of carbapenem resistant *P*. *aeruginosa* from environmental samples

Effluents from both WWTPs were sampled monthly for one-year period. Effluent water was refrigerated until further processed in the laboratory, usually within 6 hours of collection.

For selective isolation of carbapenem-resistant *P*. *aeruginosa* 100 ml of effluent water was filtered through 0.45 μm filter (Whatman). Filters were plated onto in-house selective medium (cetrimide agar (Merck)) supplemented with imipenem with a final concentration of 4 μg/mL) and incubated at 42°C for 48-hours.

After incubation up to ten suspected *P*. *aeruginosa* colonies were subcultured and identified by MALDI-TOF. All isolates confirmed as *P*. *aeruginosa* were submitted for identical antibiotic susceptibility testing as described above for clinical strains.

Isolates of *P*. *aeruginosa* resistant to either imipenem or meropenem or both were stored at -70°C for further processing.

### Genotyping by pulsed-field gel electrophoresis

All carbapenem-resistant *P*. *aeruginosa* isolates were genotyped by pulsed-field gel electrophoresis (PFGE) after *Spe*I restriction. Briefly, isolates were grown overnight on blood agar plates. The cells were resuspended in cell suspension buffer (0.18 M NaCl, 10 mM Tris, pH 8.0) to a density of 2.5 McFarland and mixed with equal volume of 1.5% agarose gel (Pulsed Field Certified^™^ Agarose, Bio-Rad prepared in buffer TE2). Cells embedded in agarose blocks were then lysed in cell lysis buffer (10 mM Tris (pH 8.0), 0.5 M EDTA, 1% (w/v) sodium dodecyl sulfate, and 0.5 mg/ml of proteinase K (Sigma)), overnight at 37°C. The DNA was digested with 10 U of *Spe*I restriction enzyme (New England Biolabs). Macrorestriction fragments were separated in 1.2% agarose gel (Pulsed field Certified^™^ Agarose, Bio-Rad) in 0.5 TBE buffer using Biometra PFGE system with the following conditions: temperature 12°C, initial switch time of 1, final switch time of 59 s, voltage of 200 V and run time 32 h. PFGE patterns were analyzed and compared using the BioNumerics software version 7.5 (Applied Maths). Clusters with ≥ 80% similarity (dendrograms were generated by UPGMA clustering method using Dice coefficient with 1.0% optimization and position tolerance) were considered to belong to the same pulsotype.

### Whole genome sequencing and MLST typing

Representative *P*. *aeruginosa* strains from each pulsotype (1 to 9 strains per pulsotype, depending on different locations and different antibiotic profile) were genotyped by multilocus sequence typing (MLST). Genomic DNA was isolated with QIAamp^®^ DNA Mini Kit (Qiagen), following the protocol for isolation of DNA from Gram-negative bacteria. Paired-end libraries were prepared with the Nextera XT sample preparation kit (Illumina) according to the manufacturer’s instructions and then run on a Miseq (Illumina) using the MiSeq^®^ Reagent Kit v3 (600 cycle). Sequence assemblies and MLST typing were performed using the SeqSphere^+^ software (Ridom GmbH). New MLST profiles were submitted to the PubMLST database (https://pubmlst.org/paeruginosa/) after which new STs were assigned. The minimum spanning tree (MST) based on MLST profiles was constructed with BioNumerics software v7.6 (Applied Maths).

### Identification of acquired carbapenemase genes

Whole genome sequences were screened for acquired carbapenemase genes using Resfinder 2.1 web-service (www.genomicepidemiology.org) [18].

### Nucleotide sequence accession number

Raw reads were submitted to the Sequence Read Archive (https://www.ncbi.nlm.nih.gov/sra) under the Bioproject accession numbers: PRJNA407721.

## Results

### Variability of carbapenem-resistant *P*. *aeruginosa* pulsotypes within clinical settings and wastewater treatment plants

**Fig 1:**
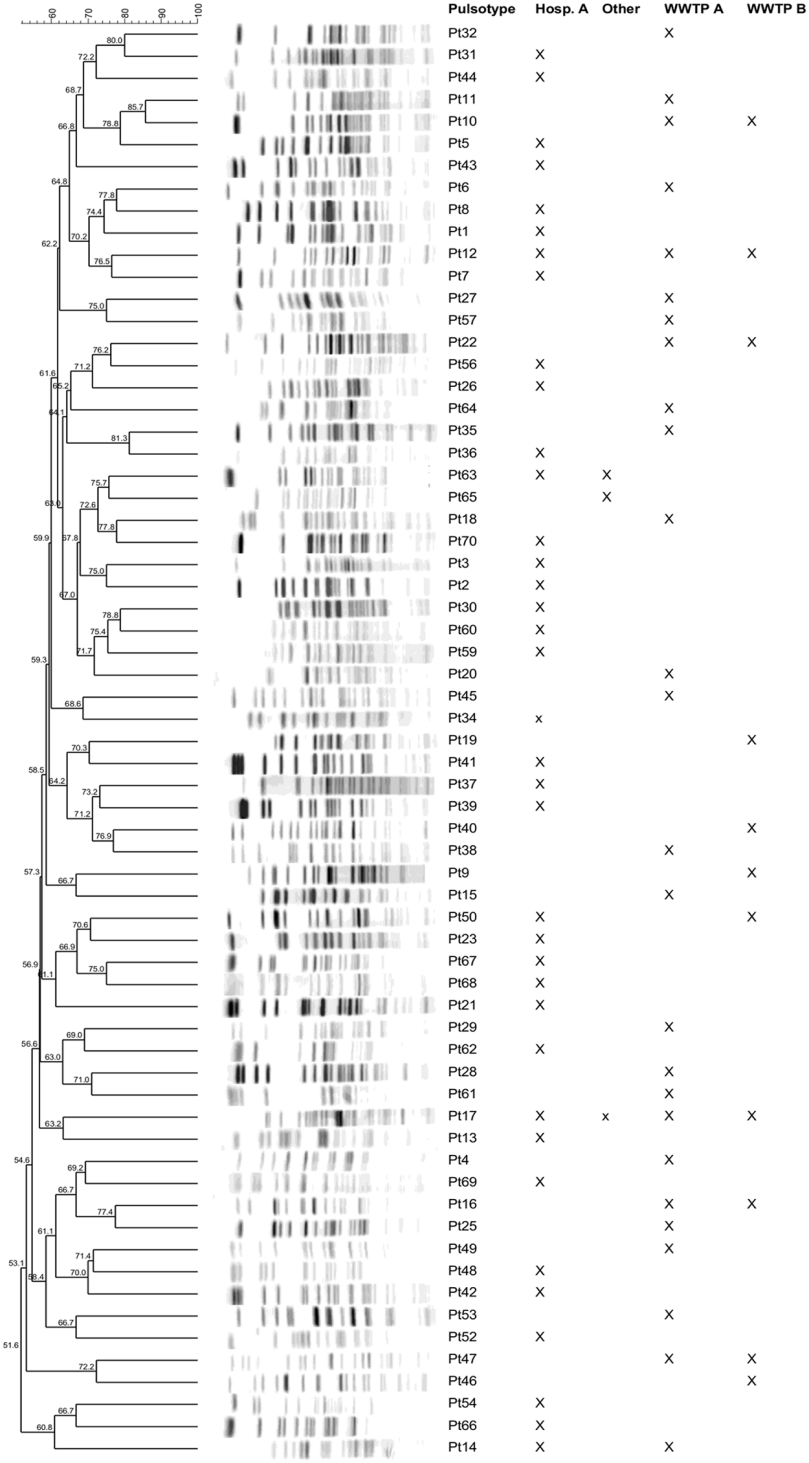
PFGE dendrogram depicting the genetic relatedness of the 66 pulsotypes found among the carbapenem-resistant *P*. *aeruginosa* isolates. Only one representative of a single pulsotype was included. Locations where the pulsotype was found are marked with the sign X. Other—other clinical settings.

Altogether 213 carbapenem-resistant *P*. *aeruginosa* were isolated from clinical and environmental samples and were classified into 65 pulsotypes (Fig 1 and Table 1).

**Table 1:** Distribution of different carbapenem-resistant *P*. *aeruginosa* pulsotypes in clinical and environmental isolates.

Pulsotypes	Total number of PFGE typed isolates	Origin of isolates			
		Clinical isolates		Environmental isolates	
		Hospital A	Other clinical settings	WWTP A	WWTP B
Pt1	57	57			
Pt10*	18			15	3
Pt17*	12	5	3 (hospital B)	2	2
Pt50*	9	8			1
Pt16*	9			6	3
Pt5	5	5			
Pt12*	5	3		1	1
Pt47*	5			3	2
Pt64	5			5	
Pt22*	4			3	1
Pt18	4			4	
Pt19	4				4
Pt2, Pt21, Pt31	3	3			
Pt63*	2	1	1 (location 1)		
Pt14*	1	1		1	
Pt40	2				2
Pt37	2	2			
Pt26, Pt30, Pt54, Pt62, Pt70	2	2			
Pt11, Pt15, Pt35,	2			2	
Pt65	1		1 (location 2)		
Pt9, Pt46,	1				1
Pt3, Pt7, Pt8, Pt13, Pt23, Pt34, Pt36, Pt39, Pt41, Pt42, Pt43, Pt44, Pt48, Pt52, Pt56, Pt59, Pt60, Pt66, Pt67, Pt68, Pt69	1	1			
Pt4, Pt6, Pt20, Pt25, Pt27, Pt28, Pt29, Pt32, Pt38, Pt45, Pt49, Pt53, Pt57, Pt61	1			1	
Total number of typed isolates	208	122	5	59	22

*—pulsotypes present in more than one setting

Clinical isolates originated from regional diagnostic laboratory serving large number of hospitals, GPs and other health care services. Carbapenem-resistant *P*. *aeruginosa* was in this time detected only in four out of eight health care facilities: a large teaching hospital (Hospital A; 1.300 beds; 55.000 discharges per year), smaller general hospital (Hospital B, 260 beds; 13.000 discharges per year) and two other health care facilities (geriatric primary care unit and psychiatric long term care facility). Air distance between hospital A and B is 22.50 km, both together service population from area of circa 2000 km^2^.

Of overall 130 carbapenem-resistant *P*. *aeruginosa* isolates obtained from 109 patients 127 isolates were typeable by PFGE and were distributed into 38 pulsotypes. From 14 patients multiple (but subsequent) isolates with different susceptibility were isolated. Samples positive on carbapenem-resistant *P*. *aeruginosa* originated from four healthcare settings, but the majority of isolates (n = 125) were from a single large teaching hospital. These isolates belonged to 37 different pulsotypes, with a single prevalent pulsotype (Pt1) which included almost half (57; 45.6%) of isolates. Other pulsotypes were represented by one to eight isolates (Table 1). From three different smaller healthcare settings only 5 carbapenem-resistant *P*. *aeruginosa* isolates belonging to 3 different pulsotypes were found (Table 1).

From WWTP effluents altogether 83 carbapenem-resistant *P*. *aeruginosa* isolates were obtained. Of these 81 were typeable and were distributed into 31 pulsotypes. Sixty-one isolates (of which 59 were typeable) originated from WWTP A, while 22 isolates originated from WWTP B. They were classified into 26 and 11 pulsotypes, respectively (Table 1). Similar to clinical isolates, also among environmental isolates a single pulsotype was prevalent (Pt10) and contained 18 isolates (21.7%). Other pulsotypes were represented by one to nine isolates (Table 1).

No prevalent pattern or uniformity of distribution of pulsotypes over the time was observed in clinical and environmental carbapenem resistant *P*. *aeruginosa* isolates (S1 and S2 Tables).

### Overlap of pulsotypes and MLST between and within clinical settings and wastewater treatment plants

**Fig 2:**
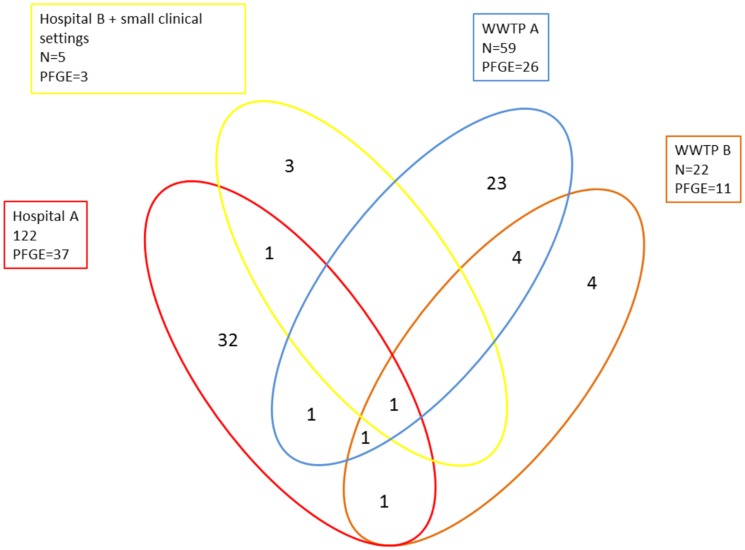
Venn diagram showing overleap of pulsotypes between clinical and environmental settings. For clearer picture and because of low number of isolates we grouped together Hospital B and smaller clinical settings. N = number of typeable isolates; PFGE = number of pulsotypes.

Within clinical isolates there were only two pulsotypes that were shared between large teaching hospital and smaller clinical settings (Pt17, Pt63) (Table 1). Isolates from the environment showed slightly higher overlap with six pulsotypes shared between both WWTPs. Overlap of pulsotypes between clinical and environmental isolates was low (Table 1, Fig 2). Only four pulsotypes were shared between clinical settings and the environment (Fig 2).

**Fig 3:**
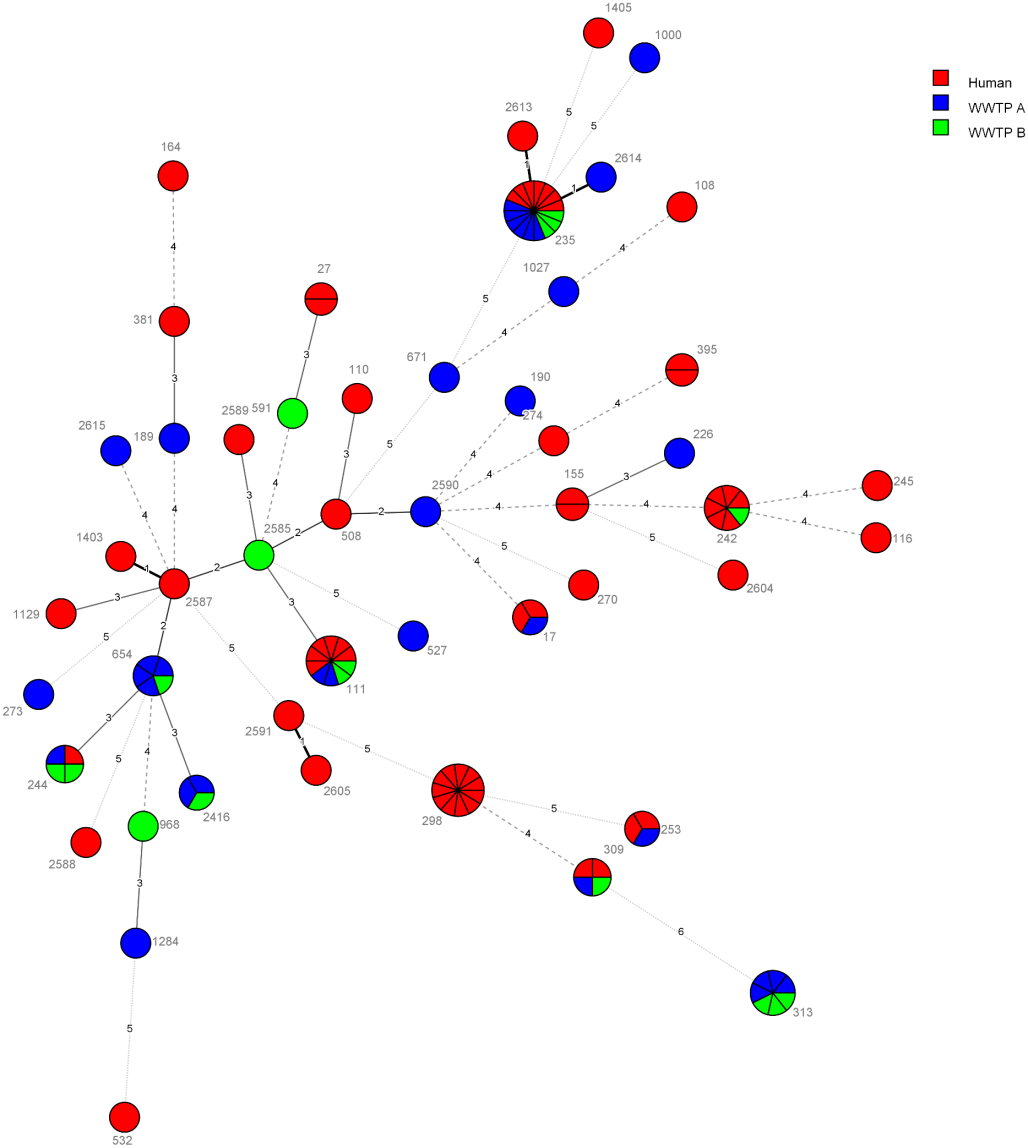
Minimum spanning tree of MLST-ST of carbapenem-resistant *P*. *aeruginosa* isolates from clinical and WWTP samples. Each circle represents one sequence type and is subdivided into sectors corresponding to the number of isolates represented with this ST. The numbers between circles represent number of differing loci between the STs. The tree is color coded according to origin.

MLST was determined for one to nine representative isolates from each pulsotype. Forty-nine different MLST types were found among 112 selected strains; 37 of them described before and 12 MLST types were new (ST-2416, -2585, -2587, -2588, -2589, -2590, -2591, -2604, -2605, -2613, -2614, -2615). No clustering or setting associated lineages (environment or clinical) were observed in minimum spanning tree (Fig 3). Correlation of MLST with all pulsotypes can be found in S3 Table.

### Antibiotic resistance of carbapenem-resistant *P*. *aeruginosa* isolates from clinical and environmental samples

Antimicrobial susceptibility of 130 isolates from 109 patients and 83 isolates from two WWTPs was tested (Tables 2 and 3). Analysis of non-redundant isolates only was assured by the initial selection of clinical isolates (see Materials and methods) and for isolates from WWTPs by including for each pulsotype only one representative isolate for each resistant pattern found per sampling (month) and per WWTP. With this approach the number of environmental isolates included in final comparative analysis narrowed from 83 to 62.

**Table 2:** Resistance to individual antibiotics.

Antibiotic	% of resistant strains (number of strains)	
	Clinical strains (N = 130^a^)	Environmental strains (N = 62^b^)
piperacilin/tazobactam	52.3 (68)	30.6 (19)
cefepime	26.2 (34)	27.4 (17)
ceftazidime	42.3 (55)	37.1 (23)
ciprofloxacin	27.7 (36)	35.5 (22)
amikacin	13.1 (17)	16.1 (10)
gentamicin	10.0 (13)	27.4 (17)
netilmicin	20.8 (27)	32.2 (20)
tobramycin	9.2 (12)	30.1 (19)

^a^—single isolate per patient,

^b^–all isolates from a single sample with identical PFGE profile and resistance profile were considered as a single strain.

**Table 3:** Resistance patterns of clinical and environmental carbapenem-resistant *P*. *aeruginosa* isolates and their corresponding pulsotypes. Pulsotypes in bold share the resistance pattern and were found in both environments (patients and water).

Group	Susceptibility pattern (resistant to)	Patients (130 strains ^a^)		WWTPs (62 strains ^b^)	
		Isolates N (%)	Corresponding pulsotypes from patients	Isolates	Corresponding pulsotypes from WWTPs
A	carbapenems	46 (35.4)	Pt1, Pt7, Pt12, **Pt14**, Pt30, Pt36, Pt37, Pt41, Pt48, Pt50, Pt54, Pt56, Pt60, Pt63, Pt66, Pt67, Pt69, Pt70	33 (53.2)	Pt2, Pt4, Pt6, Pt9, **Pt14**, Pt15, Pt16, Pt18, Pt19, Pt20, Pt22, Pt25, Pt27, Pt28, Pt32, Pt35, Pt38, Pt40, Pt46, Pt49, Pt53, Pt57, Pt61, Pt64
B	carbapenems (some but not all beta-lactams)	28 (21.5)	Pt1, Pt2, Pt21, Pt43, **Pt50**, Pt54, Pt59, NT(n = 1)	4 (6.4)	Pt16, Pt18, **Pt50**, Pt64
C	carbapenems (all other beta-lactams)	9 (6.9)	Pt1, Pt2, Pt17, Pt42, Pt50, Pt52,	0 (0)	
D	Carbapenems (at least one of the other beta-lactams), quinolones	8 (6.1)	Pt1, Pt13, Pt21, Pt30, Pt39,	0 (0)	
E	Carbapenems, quinolones	4 (3.1)	Pt1, Pt2, Pt8, Pt30	0 (0)	
F	Carbapenems, at least one of the aminoglycosides	5 (3.8)	**Pt12**, Pt37, Pt63, Pt62	3 (4.8)	**Pt12**, Pt18, Pt45
G	carbapenems, some but not all beta-lactams, at least one of the aminoglycosides	6 (5.0)	Pt3, Pt5, Pt17, Pt21, Pt23, Pt68	0 (0)	
H	Carbapenems, all other beta-lactams, quinolones, (susceptible to aminoglycosides)	7 (5.4)	Pt1, Pt34, Pt44, Pt50, Pt70	3 (4.8)	Pt47
I	carbapenems, quinolones and at least one of the aminoglycosides	2 (1.5)	Pt31, Pt65	0 (0)	
X	Carbapenems, at least one of the other beta-lactams, quinolones, at least one of the aminoglycosides	18 (13.8)	Pt5, Pt26, Pt31, **Pt17**, Pt30, NT(n = 2)	19 (30.6)	Pt10, Pt11, Pt12, **Pt17**, Pt47, Pt29, Pt58, NT (n = 1)

^a^—single isolate per patient,

^b^–all isolates from a single sample with identical PFGE profile and resistance profile were considered as a single strain,

NT—non-typeable isolate

A high percentage of resistance to piperacillin/tazobactam (52.3%) and ceftazidime (42.3%) among clinical isolates, and to ceftazidime (37.1%) and ciprofloxacin (35.5%) among environmental isolates, was found for individual antibiotics (Table 2).

We have classified strains into groups according to different resistant patterns (Table 3). The majority of isolates were resistant only to imipenem and/or meropenem (47/130 clinical isolates and 32/61 environmental isolates). Second largest group was the one with resistance to four different antibiotic classes (group X, Table 3). Clinical isolates were found in all groups, while environmental isolates were found only in 5 groups with the greatest proportion in group A (resistant to carbapenems only) and group X (resistant to all classes of antibiotics tested). Isolates with the same pulsotype and same MLST-ST can have a different resistance pattern.

### Identification of acquired carbapenemase resistance genes

We have screened 112 *P*. *aeruginosa* genomes with Resfinder and found two types of acquired carbapenemase genes in 18 genomes. Gene *bla*_VIM-2_ was present in three MLST-ST types (ST111/Pt17; ST235/Pt10, Pt11, Pt3; ST654/Pt47, Pt18). Gene *bla*_VIM-1_was present only in ST235. Strains with carbapenemase genes were isolated from hospitals A and B and from both WWTPs.

Most strains carrying carbapenemase have been classified into resistant pattern group X (15/18), three of the rest was in group H or G respectively.

## Discussion

In this study, we assessed variability and antibiotic resistance profiles of carbapenem-resistant *P*. *aeruginosa* isolated from different clinical settings and two WWTPs. In contrast to other studies comparing environmental and clinical isolates which focused on pseudomonads or *P*. *aeruginosa* in general [15,19–22] we included in the analysis only carbapenem-resistant isolates of *P*. *aeruginosa*. Similar as reported for entire *P*. *aeruginosa* population, the diversity of selected carbapenem-resistant *P*. *aeruginosa* isolates, including clinical and environmental isolates, was also high.

Of 65 pulsotypes only 9 were shared between two or more settings (hospital or WWTP) (Fig 2). In these nine pulsotypes we found 10 different MLST-STs. Some of them, ST111 and ST235, are also the most successful clones with respect to worldwide dissemination of resistant *P*. *aeruginosa* strains [23–25]. Some others were described before, but seem to be limited to certain geographic areas [24,26–28]. One MLST type was not yet reported (ST2416).

Low level of overlap between clinical and environmental isolates observed in this study is in line with previous reports [20,29,30]. Despite this low overlap, the MLST minimum spanning tree does not show any lineage to be environment or clininc specific. In our study the overlap of genotypes between the two WWTPs seems to be higher than between the clinical settings (Figs 2 and 3). Only one of the first three most prevalent pulsotypes are shared between environment and clinical isolates (Table 1). Interestingly, some pusotypes only occasionally detected in hospitals were shared between hospitals and WWTPs, while the most prevalent pusotype in the study (Pt1) was found only in hospital A during entire 12 months study interval and never in WWTPs. The reason for this is unclear.

Some studies support hypothesis that antibiotic resistance would be more common in hospital strains [21,31–33], others showed the opposite and described higher percentage of resistant *P*. *aeruginosa* strains in the environment. High percentage of resistant *P*. *aeruginosa* strains in the environment was for example described in swimming pools [34], untreated hospital wastewater [35] and wastewater effluent [15]. These resistant environmental isolates are mostly not genetically related to clinical isolates, but they might still serve as a potential reservoir of determinants for carbapenem resistance [36,37]. In our study, focused only on carbapenem resistant *P*. *aeruginosa*, there was no clear distinction in the proportion of resistant strains among clinical or environmental strains, but clinical isolates showed higher diversity in resistance patterns (Table 3). Coexistence of different resistance patterns within the same pulsotype and MLST-type was observed, similarly to findings by Gomilla and colleagues, who found resistant and nonresistant isolates in same MLST- STs [38]. Pulsotype Pt17 (ST111), which was the only one shared between more than 3 settings, included isolates from 4 different resistance groups. All environmental isolates, sharing the same pulsotypes with clinical isolates, had also same resistant patterns than some of clinical isolates of that pulsotypes (Table 3). Predominant pulsotypes were distributed throughout the defined resistance patterns. The most prevalent pulsotype, Pt1, displayed 5 out of 10 resistance patterns but not the most complex one (group X; Table 3). On the contrary, second most often isolated pulsotype, Pt10, was present only in this group (group X).

Specific acquired carbapenemase genes were found only in 18 of 112 tested carbapenemase resistant *P*. *aeruginosa* strains from 3 STs and 6 pulsotypes. Strains were isolated from hospitals and from WWTPs. All of them were VIM-type metallo-beta-lactamases of class B, mostly VIM-2. Currently, VIM-2 is the most widespread metallo-beta-lactamase in *P*. *aeruginosa* (39). All three STs found in our study (ST111, ST235, ST654) are associated with multidrug resistant *P*. *aeruginosa* strains in clinical settings [39,40].

In conclusion, we show here that carbapenem-resistant *P*. *aeruginosa* are ubiquitous in WWTP effluents and that they represent a diverse population of carbapenem-resistant *P*.*aeruginosa* compared clinical strains. In spite of that we should be aware that environment can be a source of successful multiresistant strains which carry acquired resistance genes.

## Supporting information

S1 Table Carbapenem-resistant *P*. *aeruginosa* pulsotypes isolated from patients showing time of isolation (month) and origin of the strain regarding institution. (PDF)

S2 Table Carbapenem-resistant *P*. *aeruginosa* pulsotypes isolated from WWTPs showing time of isolation (month) and origin of strain regarding WWTP A or WWTP B. (PDF)

S3 Table MLST sequence types determinated from different pulsotypes. (PDF)
