# Supplementary material for: Low overlap between carbapenem resistant Pseudomonas aeruginosa genotypes isolated from hospitalized patients and wastewater treatment plants
Source: PLoS One. 2017 Oct 19;12(10):e0186736. doi: 10.1371/journal.pone.0186736 (PMC5648238; doi:10.1371/journal.pone.0186736)
Supplement: S1 Table — (PDF) [file pone.0186736.s001.pdf]

**Supplementary table 1.** Carbapenem-resistant *P. aeruginosa* pulsotypes isolated from patients showing time of isolation (month) and origin of the strain regarding institution

| Pulsotype | Number of isolates | I. | II. | III. | IV. | V. | VI. | VII. | VIII. | IX. | X. | XI. | XII. | Hospital A | Hospital B | Small clinical settings |
|---|---|---|---|---|---|---|---|---|---|---|---|---|---|---|---|---|
| Pt1 | 57 | 3 | 6 | 8 | 8 | 6 | 4 | | 3 | 3 | | 11 | 5 | X | | |
| Pt17 | 8 | | 1 | | | 1 | 1 | | | 1 | | 2 | 2 | X | X | |
| Pt50 | 8 | | | 3 | 3 | | | 1 | | | 1 | | | X | | |
| Pt5 | 5 | | | | | | | | | 2 | 1 | | | X | | |
| Pt2 | 3 | | | | | 2 | | | | | | 1 | | X | | |
| Pt12 | 3 | | | | 1 | | 1 | | | | | | 1 | X | | |
| Pt21 | 3 | | | 1 | | | | | | | 1 | 1 | | X | | |
| Pt31 | 3 | | | | | | | | | | | 3 | | X | | |
| Pt26 | 2 | | | | 1 | | 1 | | | | | | | X | | |
| Pt30 | 2 | | | | | | | | | | | 1 | 1 | X | | |
| Pt54 | 2 | | 2 | | | | | | | | | | | X | | |
| Pt62 | 2 | | | | | | | 1 | | | 1 | | | X | | |
| Pt63 | 2 | | 1 | | | | | | | 1 | | | | X | | X |
| Pt3 | 1 | | | | | | | | | | | 1 | | X | | |
| Pt7 | 1 | | | | | | 1 | | | | | | | X | | |
| Pt8 | 1 | | | | | | | | | | | 1 | | X | | |
| Pt13 | 1 | | | | | | | | 1 | | | | | X | | |
| Pt14 | 1 | | | | | | | | 1 | | | | | X | | |
| Pt23 | 1 | | | | | | | | | | 1 | | | X | | |
| Pt34 | 1 | | | 1 | | | | | | | | | | X | | |
| Pt36 | 1 | | | 1 | | | | | | | | | | X | | |
| Pt37 | 1 | | | | | | | | | | | | 1 | X | | |
| Pt39 | 1 | | | | | | | | | | 1 | | | X | | |
| Pt41 | 1 | 1 | | | | | | | | | | | | X | | |
| Pt42 | 1 | | | | | 1 | | | | | | | | X | | |
| Pt43 | 1 | | | | | | | | | 1 | | | | X | | |
| Pt44 | 1 | | | | 1 | | | | | | | | | X | | |
| Pt48 | 1 | | | | | 1 | | | | | | | | X | | |
| Pt52 | 1 | | | | 1 | | | | | | | | | X | | |
| Pt56 | 1 | | | | | | | | | | | 1 | | X | | |
| Pt59 | 1 | | | | | | | | | | | | | X | | |
| Pt60 | 1 | | | | | | | | | | 1 | | | X | | |
| Pt66 | 1 | | | | | | | | | 1 | | | | X | | |
| Pt65 | 1 | | | | | | | 1 | | | | | | | | X |
| Pt67 | 1 | | | | | | | | 1 | | | | | X | | |
| Pt68 | 1 | | | | | | | | | | 1 | | | X | | |
| Pt69 | 1 | | | | | | | | | | | 1 | | X | | |
| Pt70 | 2 | | | 2 | | | | | | | | | | X | | |
