# Supplementary material for: Low overlap between carbapenem resistant Pseudomonas aeruginosa genotypes isolated from hospitalized patients and wastewater treatment plants
Source: PLoS One. 2017 Oct 19;12(10):e0186736. doi: 10.1371/journal.pone.0186736 (PMC5648238; doi:10.1371/journal.pone.0186736)
Supplement: S2 Table — (PDF) [file pone.0186736.s002.pdf]

Supplementary table 2. Carbapenem-resistant *P. aeruginosa* pulsotypes isolated from WWTPs showing time of isolation (month) and origin of strain regarding WWTP A or WWTP B

Pulsotype	Number of isolates	I.	II.	III.	IV.	V.	VI.	VII.	VIII.	IX.	X.	XI.	XII.	WWTP A	WWTP B
Pt10	18			1	1	4	2	7	1			1	2	X	X
Pt16	9	1				1			4			1	2	X	X
Pt47	5			1				1	2	1				X	X
Pt64	5				1	3	1							X	
Pt17	4			1			1	1		1				X	X
Pt18	4			1		1					1		1	X	
Pt19	4										4				X
Pt22	4			1		1	2							X	X
Pt11	2			2										X	
Pt12	2			1								1		X	X
Pt15	2										2			X	
Pt35	2			1							1			X	
Pt40	2										2				X
Pt4	1									1				X	
Pt6	1												1	X	
Pt9	1	1													X
Pt14	1										1			X	
Pt20	1											1		X	
Pt25	1						1							X	
Pt27	1						1							X	
Pt28	1						1							X	
Pt29	1			1										X	
Pt38	1						1							X	
Pt45	1											1		X	
Pt46	1										1				X
Pt49	1									1				X	
Pt50	1					1									X
Pt53	1							1						X	
Pt57	1					1								X	
Pt61	1				1									X	
Pt32	1										1			X	
