# Supplementary material for: Low overlap between carbapenem resistant Pseudomonas aeruginosa genotypes isolated from hospitalized patients and wastewater treatment plants
Source: PLoS One. 2017 Oct 19;12(10):e0186736. doi: 10.1371/journal.pone.0186736 (PMC5648238; doi:10.1371/journal.pone.0186736)
Supplement: S3 Table — (PDF) [file pone.0186736.s003.pdf]

Supplementary table 3: MLST sequence types determinated from different pulsotypes

			Origin of isolates			
			Clinical Isolates		Environmental isolates	
Pulsotypes	Total number of sequenced isolates	MLST type	HOSPITAL A	OTHER CLINICAL SETTINGS	WWTP A	WWTP B
Pt1	9	298	57			
Pt2	2	17	3			
Pt3	1	235	1			
Pt4	1	190			1	
Pt5	4	235	5			
Pt6	1	189			1	
Pt7	1	155	1			
Pt8	1	2605	1			
Pt9	1	968				2
Pt10	6	235 (5 genomes) 313 (1 genome)			15	3
Pt11	1	235			2	
Pt12	4	309	3		1	1
Pt13	1	245	1			
Pt14	2	253	1		1	
Pt15	1	313			2	
Pt16	4	313	9		6	3
Pt17	7	111	5	3	3	3
Pt18	2	654			4	
Pt19	2	244				4
Pt20	1	244			1	
Pt21	1	108	3			
Pt22	3	2416			3	1
Pt23	1	2588	1			
Pt25	1	17			1	
Pt26	2	381(1 genome) 27(1 genome)	2			
Pt27	1	235			1	
Pt28	1	2590			1	
Pt29	1	671			1	
Pt30	1	270	2			
Pt31	2	111	3			
Pt32	1	235			1	
Pt34	1	116	1			

Pt35	1	1027	2		2	
Pt36	1	532	1			
Pt37	1	395	2			
Pt38	1	527			1	
Pt39	1	2589	1			
Pt40	1	2585				2
Pt41	1	2587	1			
Pt42	1	1403	1			
Pt43	1	155	1			
Pt44	1	2591	1			
Pt45	1	273			1	
Pt46	1	591				1
Pt47	4	313(1 genome)<br>654(1 genome)	5		3	2
Pt48	1	110	1			
Pt49	1	2615			1	
Pt50	5	242	8			1
Pt52	1	242	1			
Pt53	1	266			1	
Pt54	2	27(1 genome)<br>298(1 genome)	2			
Pt56	1	298	1			
Pt57	1	2614			1	
Pt59	1	1405	1			
Pt60	1	244	1			
Pt61	1	1000			1	
Pt62	1	2604	2			
Pt63	2	508(1 genome)<br>274(1 genome)	1	1		
Pt64	1	1284			5	
Pt65	1	111		1 (location 2)		
Pt66	1	253	1			
Pt67	1	298	1			
Pt68	1	242	1			
Pt69	1	164	1			
Pt70	2	235(1 genome)<br>1129(1 genome)	2			
